# Brain Structure and Function in Recovery

**DOI:** 10.35946/arcr.v40.3.04

**Published:** 2020-12-03

**Authors:** Sara Jo Nixon, Ben Lewis

**Affiliations:** 1 Department of Psychiatry, University of Florida, Gainesville, Florida; 2 Center for Addiction Research & Education, University of Florida, Gainesville, Florida

**Keywords:** alcohol, alcohol use disorder, neurobehavioral deficits, brain structure, neurophysiology, neurochemistry, recovery, neural networks

## Abstract

Alcohol use disorder (AUD) commonly is associated with compromise in neurobiological and/or neurobehavioral processes. The severity of this compromise varies across individuals and outcomes, as does the degree to which recovery of function is achieved. This narrative review first summarizes neurobehavioral, neurophysiological, structural, and neurochemical aberrations/deficits that are frequently observed in people with AUD after detoxification. Subsequent sections review improvements across these domains during recovery, taking into account modulators of recovery to the extent permitted. Where appropriate, the discussion includes work integrating outcomes across domains, leveraging the strengths of diverse experimental methods. Interventions to ameliorate neurobiological or neurobehavioral deficits do not constitute a primary objective of this review. However, their consideration is a logical inclusion. Therefore, a limited introduction to existing methods is also presented.

## INTRODUCTION

Alcohol use disorder (AUD) is characterized by dysregulation across a range of neurobiological and/or neurobehavioral domains. Neurobiological aberrations include dysregulated neural activity and patterns of brain activation as well as compromise in gray and white matter. Neurobehavioral aberrations are widespread and evident across diverse neuropsychological domains such as problem-solving, learning, memory, and motor functions. An estimated 50% to 80% of people with AUD demonstrate significant cognitive/behavioral compromise relative to community comparison groups, with a substantive minority (i.e., 30% to 40%)^1^ exhibiting sufficient compromise to meet criteria for clinical impairment.^2^ Describing alcohol-related impairment is further complicated by the fact that neurobiological (e.g., structural) aberrations and behavioral compromise are not universally related. Importantly, empirical studies demonstrate that both neurobiological and behavioral measures improve substantially after recovery is initiated, although the trajectories vary and are often incomplete. This narrative review focuses on improvements in brain structure and function and briefly explores opportunities for facilitating these processes. To establish an appropriate context, the article begins with a limited overview of alcohol-related biobehavioral deficits. More comprehensive coverage of alcohol-related impairment is provided in several recent reviews.^3,4^

In discussing recovery, several caveats warrant attention. First, there is a paucity of data from individuals who address their alcohol misuse without seeking formal treatment. Thus, this review is largely limited to outcomes obtained from people who participated in inpatient or intensive outpatient treatment.

Second, the phrase “in recovery” eludes ready definition. The goals of both the individuals with AUD and the treatment programs vary. If a program is abstinence based, the objective is to sustain abstinence after treatment, and an individual is considered “in recovery” as long as they maintain abstinence. If the primary treatment objective is harm reduction or controlled drinking, successful recovery is marked by a reduction in negative consequences, without abstinence as a necessary prerequisite. Consequently, while both people who sustain abstinence and those who successfully navigate harm reduction efforts can be considered “in recovery,” their continuing exposure to alcohol may vary significantly. Thus, heterogeneity in continued drinking across studies creates a substantive interpretational challenge, prohibiting broad conclusions regarding the effects of “recovery” on neurobiobehavioral improvement. To address this challenge, studies need to incorporate alternate definitions of “successful” outcomes, perhaps also including neurobiobehavioral improvement as one component. In the extant literature, the majority of reports are derived from treatment-seeking individuals in abstinence-based programs. Nevertheless, rather than relying only on binary outcomes (e.g., relapse vs. sustained abstinence), some investigations, as illustrated in later sections, gather data regarding continuing drinking patterns, providing a more granular consideration of alcohol use across time.

Third, many studies use the phrases “recovery” and “improvement” of function interchangeably. At initial glance, distinguishing these terms seems a matter of semantics. However, as addiction science directs attention to the effectiveness of interventions in enhancing outcomes, the distinction is highly relevant.^2^ Conservatively defined, improvement references positive change associated with the passage of time (i.e., time-dependent change) or repeated practice (i.e., practice effects). For example, cognition improves with time after detoxification, even without directed intervention, as well as after repeated testing. The phrase “recovery of function,” in contrast, refers to positive change that cannot be accounted for by time or practice. Distinguishing “improvement” from “recovery” requires the inclusion of appropriate comparison data and is particularly relevant when evaluating behavioral outcomes and interventions. In the following sections, the terms are used with attention to this distinction. That said, positive change is a desired outcome, whether or not it meets a strict definition of recovery of function.

Fourth, although the potential influence of individual variables such as age and sex/gender on recovery is widely recognized, it has not been systematically studied, particularly in longitudinal assessments. Therefore, these variables are not discussed in depth here.

## BRIEF OVERVIEW OF ALCOHOL-RELATED SEQUELAE

This section provides brief overviews of four broad categories of alcohol-related biobehavioral sequelae: neurobehavior, neurophysiology, brain structure, and neurochemistry.

### Neurobehavior

A substantial literature has illustrated that cognitive processes relying heavily on the prefrontal and frontal cortices (i.e., executive functions such as attention, working memory, problem-solving, inhibition, and flexibility) are susceptible to chronic excessive alcohol consumption.^5^ However, alcohol-related deficits are not limited to these domains. Compromise in visual-spatial functions, gait/balance, and new learning/memory is also frequently reported.^6^ Taken together, alcohol-related deficits in neuropsychological/behavioral functions often are described as reflecting a mild, generalized brain dysfunction.^2,6^ Beyond these traditional neuropsychological characteristics, interest in alcohol-related compromise in key facets of emotion processing and social cognition is increasing. Of particular note are deficits in emotion face processing, interpersonal problem solving, and humor processing,^3,7,8^ all of which are critical skills in social, work, and family settings.

### Neurophysiology

Brain electrophysiology, as obtained from scalp electrodes, also is affected by chronic alcohol misuse. Studies have revealed dysregulation in the electroencephalogram (EEG), as well as in the amplitudes and/or latencies of electrophysiological components that occur at specific times following stimulus presentation or response (i.e., event-related potentials [ERPs]).^9,10^ Importantly, both ERP components that occur earlier after stimulus presentation (i.e., exogenous components) and reflect sensory processes and components that occur later (i.e., endogenous components) and reflect cognitive processes are sensitive to chronic excessive alcohol use. This demonstrates alcohol’s impact on the temporal dynamics of both sensory and cognitive processes.^7,9,10^ A growing body of alcohol research has focused on performance monitoring, which entails ongoing monitoring of response accuracy in the context of changing demands. A common variable studied in these protocols is the error-related negativity (ERN), which is observed after the subject commits an error while completing speeded response tasks.^11^ Accurately detecting errors is essential for adaptive behavior. Thus, findings of aberrant ERN amplitudes in people with AUD^12^ suggest compromise in the biobehavioral dynamics underlying adaptive behavior.

Repetitive patterns of neural activity (i.e., neural oscillatory activity) and the amount of brain activity in certain frequency bands (i.e., EEG power) reflect a coordinated (i.e., synchronous) neuronal discharge that can be examined as a function of both time and frequency. EEG power can be examined in either a resting state or during specific sensory or cognitive events. In the latter case, the activity is referred to as event-related oscillations. AUD is associated with alterations in both types of measures, demonstrating widespread dysregulation in the temporal dynamics of neural processes.^10^

### Brain Structure

People with AUD frequently exhibit volumetric loss in gray and white matter, as well as ventricular expansion in both the cerebrum and cerebellum.^13,14^ Data regarding sex differences are mixed, with some studies suggesting that women are more susceptible than men to alcohol’s effects while other studies show either no pattern or the opposite pattern.^15^ Higher vulnerability also has been reported with increasing age, especially in frontal brain areas.^16^ Beyond reduced brain volumes, studies have shown compromised white matter integrity,^17,18^ with indications of age interactions.^19^

Dysregulation in brain network activity and connectivity also frequently occurs.^20^ Although the default mode network^21^ has received greatest attention, other networks are impacted as well, including the executive control, salience, and reward networks.^22,23^ Finally, associations may exist between structural compromise and neurobehavioral measures. For example, Pandey and colleagues^18^ found significant relationships between white matter fractional anisotropy measures and neuropsychological performance.

### Neurochemistry

Several studies have demonstrated that neurochemistry is also disrupted in AUD.^24,25^ Using proton magnetic resonance imaging, the most frequently reported findings indicate lower levels of the neuronal metabolite *N*-acetylaspartate (NAA), as well as of choline-containing compounds (Cho) and creatine metabolites (Cr). Findings are mixed regarding alcohol’s effects on the glial metabolite myo-inositol, and complex outcomes are associated with measures of the neurotransmitters glutamate and gamma-aminobutyric acid (GABA).^26^

### Summary

Although they do not occur in all people with AUD, alcohol-related deficits in neurobehavior, neurophysiology, brain structure, and neurochemistry constitute significant individual and public health concerns. Deficits across the four domains are incompletely correlated and often fall short of criteria for clinical impairment. Nevertheless, they can impact treatment engagement, post-treatment adaptation, and relapse.^27–30^ Thus, clarifying recovery trajectories, identifying relevant individual and confounding variables, and determining effective interventions must be research priorities.

## EFFECTS OF RECOVERY

Fortunately, with continuing recovery, neurobiobehavioral impairment can improve. The following sections discuss neurobehavioral, neurophysiological, structural, and neurochemical recovery in more detail.

### Neurobehavioral Change in Recovery

Investigations suggest that substantial improvements in neurobehavioral functions occur during the first 4 to 8 weeks of abstinence, followed by more modest mid-term (i.e., approximately 1 year) gains. Verbal skills typically improve most quickly, while other domains, although improved, may remain compromised for several months to years.^31^ Longitudinal studies also found substantive differences in change trajectories across domains, supporting the general conclusions derived from cross-sectional comparisons of subgroups of people with AUD who differed in abstinence length.^4^ Petit and colleagues^32^ recently investigated the effects of abstinence on alcohol-related working memory and inhibitory control deficits. By the third week of abstinence, working memory function was indistinguishable between the AUD and control groups, whereas inhibitory control deficits remained. Employing a similar 3-week test interval, Cordovil De Sousa Uva and colleagues^33^ also observed deficits in inhibitory control and executive functions at initial testing, but noted no improvements at retest for either function. Not surprisingly, recovery across these three overarching domains appears to be greatest with abstinence.^27,34–36^ However, it is noteworthy that some data suggest that low or moderate posttreatment drinking may not preclude improvement.^29^

Studies of improvement in cerebellum-linked behaviors such as gait, balance, and postural sway have produced mixed results. Fein and Greenstein^37^ examined these functions in a longitudinal study of people with AUD, with a baseline assessment at 6 to 15 weeks of abstinence and follow-up 4 to 16 months later. Performance was compared with healthy control subjects who also were tested twice. The AUD group performed more poorly than the control group at both assessments and demonstrated no improvement across time. The investigators note that the analyses would have missed improvement occurring before the first assessment (i.e., an average of about 10 weeks of abstinence). However, persistence of deficits in cerebellar functions also has been demonstrated in other studies and in both men and women.^38^ To date, most studies on the recovery of alcohol effects on the cerebellum have been restricted to measures of stability and related outcomes. This focus is expected to expand with increasing appreciation of the cerebellum’s role in extended brain networks.^39,40^

Research regarding initial deficits as well as recovery in social cognition is limited and has yielded mixed results,^3^ but recent work provides encouragement. For example, Erol and colleagues^41^ observed improvements in emotion identification accuracy, with performance in people with AUD at 3 months of abstinence equivalent to that of control subjects. It is possible that improvement in emotion processing and social cognition may require more time than do more commonly investigated cognitive functions.

One limitation of these studies is that AUD-focused longitudinal examinations often assess participants only at two time points and typically within a relatively narrow time frame to minimize participant attrition and ensure study feasibility. This practice significantly constrains understanding of continued recovery and limits estimations of within-person heterogeneity, minimizing the opportunity to identify differential predictors and trajectories at the level of the individual. A study by Bates and colleagues^42^ provides a notable exception, revealing marked within-person heterogeneity and illustrating substantive challenges in predicting recovery trajectories.

**Figure 1:**
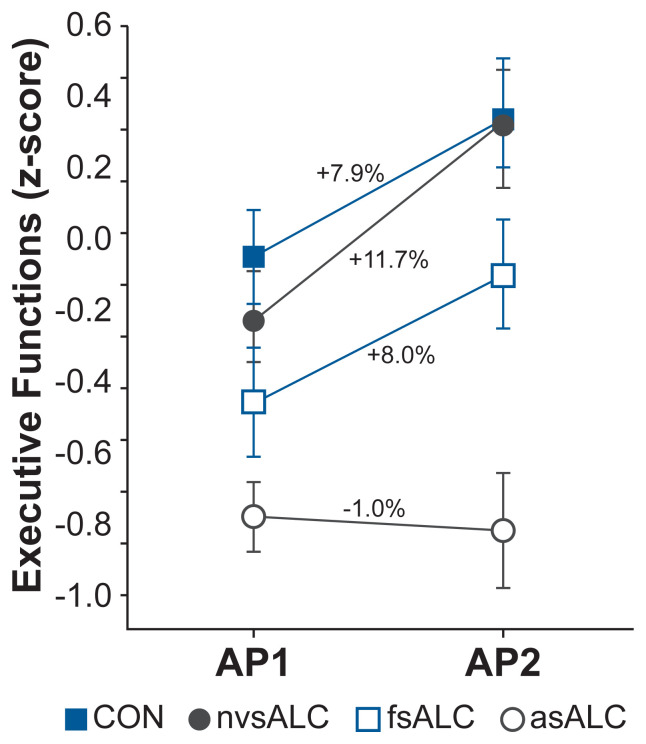
Effect of smoking status on recovery of executive functions during abstinence Over an 8-month post-treatment period, individuals with alcohol use disorder (AUD) who never smoked evinced greater improvement in executive functions (as indicated by z-score) relative to all other groups. Active smokers showed no improvement between assessments, remaining inferior to controls and people who never smoked. The slight increase in the control group could be expected based on practice effects. *Note:* AP1: 33 ± 9 days abstinent; AP2: 232 ± 56 days abstinent; CON: never-smoking controls; nvsALC: never-smoking individuals with AUD; fsALC: former smokers with AUD; asALC: active smokers with AUD. *Source:* Durazzo and Meyerhoff, 2020.^44^ Reprinted with permission from Elsevier Inc.

Nicotine use, particularly chronic smoking, is common in people seeking treatment for AUD. Several studies have examined its potential role in exacerbating alcohol-related deficits. Durazzo and colleagues^34^ compared recovery trajectories across an 8-month assessment period in active smokers and nonsmokers with similar initial deficits. Whereas the nonsmokers demonstrated recovery of cognitive function, the active smokers retained measurable deficits on multiple measures. Age played a significant role in this relationship, with older active smokers evincing the least improvement over time.^43^ In a recent follow-up study, Durazzo and Meyerhoff^44^ compared people with AUD who were either never smokers (nvsALC), former smokers (fsALC), or active smokers (asALC) with a healthy control group. All participants were tested twice: The AUD groups were assessed at about 30 days of abstinence and again at about 8 months of sustained abstinence, and the control group was tested and retested at a similar interval. In contrast to earlier work focusing on learning/memory,^34^ the researchers administered a more comprehensive battery. Smoking status accounted for differential recovery across all neurocognitive domains, including executive functions (see Figure 1), with active smokers exhibiting the least recovery.

### Neurophysiological Change in Recovery

The degree to which brain electrophysiology improves with abstinence is variable and influenced by family history of AUD. For example, seminal studies showed that components of early sensory potentials, such as the brainstem auditory evoked response, exhibited improved morphology, shortened conduction times, and shorter latencies at 4 months of abstinence than at 1 month of abstinence.^9^ In contrast, amplitudes for the P3—a later component associated with context (target) processing, cognitive control, and feedback processing—remained dampened. Importantly, a family history of AUD accounted for much of the variability in P3 amplitude. Similar observations across numerous studies have led to the proposal that P3 aberrations, particularly blunted P3 amplitudes, constitute a possible AUD endophenotype.^10,45,46^

Using a cross-sectional design, Fein and colleagues^47^ investigated the effect of abstinence on neurobiological variables, comparing individuals with AUD who were long-term abstinent (abstinence ≥ 6 months, mean abstinence > 6 years) and community controls. The investigators examined the P160—an ERP component with demonstrated sensitivity to face processing and reaction time—using an emotional face expression task. In this task, individuals must select the emotion expressed by individually presented faces. The control task required identifying a neutral face as either male or female. Compared with the community controls, the long-term abstinent group demonstrated longer P160 latencies on both tasks and slower reaction times on the emotional face expression task only. The P160 effects remained significant even after accounting for reaction-time differences. In contrast to other work,^9,10^ family history of AUD did not influence outcomes in the current study. Also, no significant sex by group interactions were observed, a finding contrary to the common conclusion that men and women are differentially vulnerable.

Several studies have used resting state synchrony (RSS) in studies of recovery. RSS reflects the level of synchrony in activation and/or deactivation within or across brain areas when an individual is not actively engaged in a neurocognitive task, i.e., at rest. Using RSS, Camchong and colleagues^35,36^ examined differences between short-term (mean = 73 days) and long-term (mean = 7.9 years) abstinence as reflected in activation patterns within the executive control and reward processing networks. They found that, when compared to community controls and individuals with short-term abstinence, individuals with long-term abstinence displayed significantly lower levels of RSS in the reward processing network than did either the short-term abstinent or community control groups. Individuals who had achieved short-term abstinence fell intermediate to the community and long-term participants, but did not differ significantly from the control participants. Longer abstinence was also associated with higher levels of RSS in the executive control network, although group comparisons indicated that only the contrast between the long-term and community groups was statistically different.

**Figure 2:**
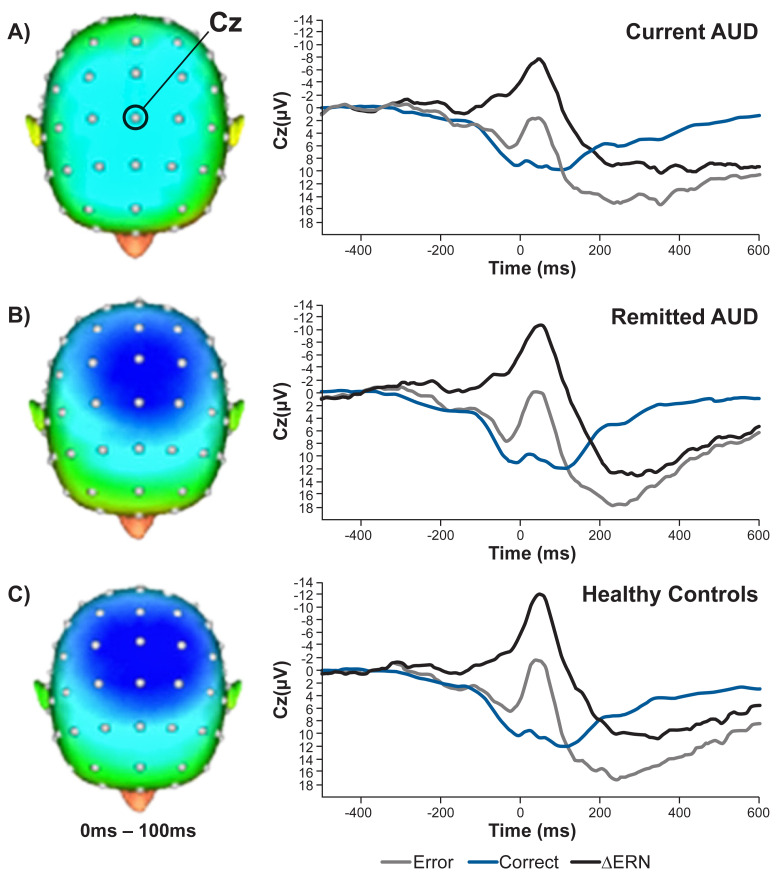
Error-related neural activity among (A) people with current AUD, (B) people with remitted AUD, and (C) healthy controls (Left) Topographic maps of neural activity (error minus correct). (Right) Response-locked event-related potential waveforms for correct trials, error trials, and difference waves (error-related negativity; ΔERN). Current AUD was associated with greater blunting of the ΔERN amplitude relative to both healthy controls (Cohen’s *d* = 0.52) and individuals with remitted AUD (Cohen’s *d* = 0.37). Individuals with remitted AUD did not differ from healthy controls. Cz: electrode located at the central midline position; ms, milliseconds. *Source:* Gorka et al., 2019.^12^ Reprinted with permission from Elsevier Inc.

Alterations in processes underlying intentional behavior likely contribute to long-term outcomes. As previously described, the ERN is an indicator of effective performance monitoring. A recent cross-sectional study examined the ERN in (a) actively drinking, non–treatment-seeking people with AUD; (b) individuals meeting criteria for remitted AUD using clinical criteria assessing drinking consequences and which do not require abstinence (mean = 2.8 years in remission); (c) individuals with a family history of AUD, but not having an AUD themselves; (d) people without histories of AUD who met criteria for non-psychotic disorders such as anxiety or depression; and (e) healthy controls.^12^ In contrast to earlier reports indicating that AUD was associated with higher ERN amplitudes,^48^ the actively drinking AUD group in this study produced significantly lower ERN amplitudes than each of the other groups, which did not differ among themselves (see Figure 2). Interestingly, there were no group differences in accuracy rate or reaction times for errors. Also, the study found no effect of a family history in the AUD groups, although prior work by Fein and Chang^49^ had indicated that an increased family-history density in people with AUD was associated with lower ERN amplitude. Regardless of the direction of the alcohol effect or the possible role of a family history of AUD, these data implicate dysregulation in neural activity in detecting behavioral errors, which is a critical aspect of effective intentional behavior.

### Structural Change in Recovery

Demirakca and colleagues^50^ studied change in gray and white matter in treatment-seeking men and women between 5 weeks and 3 months of post-treatment abstinence. They found a significant reduction in cerebral spinal fluid (CSF), an indicator of ventricular enlargement and significant increases in gray matter volume, particularly in the insula and cingulate gyrus, for participants who sustained abstinence over the interim period. In contrast, participants who used alcohol, regardless of the amount, demonstrated no change. Unfortunately, the sample size was insufficient to address potential sex differences. Another study compared imaging analyses of treatment-seeking individuals with AUD and healthy controls on day 1 and day 14 of treatment.^51^ The treatment group showed significant, but incomplete, recovery in gray matter volume even across the limited time frame, with the cingulate gyrus, temporal gyrus, parietal lobule, cerebellum, and precuneus exhibiting greater improvement than other areas examined. A preliminary examination of sex differences revealed no sex by group interactions, suggesting the absence of sex differences in the trajectory of this measure of brain recovery.

Another longitudinal study examined structural changes over a 6-month period.^29^ Rather than using a binary classification of outcomes (i.e., sustained abstinence vs. return to alcohol use), the investigators quantified alcohol use across the study period. The analyses indicated an inverse relationship between consumption across the 6 months and volume increases in diverse brain regions, including the cerebellar vermis, fusiform gyrus, striatum, and cingulate gyrus. The pattern of this association suggested that measurable brain volume improvement may be observed with low to moderate alcohol use after treatment, at least over this 6-month period. However, the small sample size dictates caution in broad generalization.

Employing longitudinal assessments of their sample, Meyerhoff, Durazzo, and colleagues conducted a series of analyses based on longitudinal assessment of individuals with AUD to address recovery trajectories. Imaging sessions at 1 week, 1 month, and 7.5 months of sustained abstinence found substantive volume increases in the frontal, parietal, and occipital lobes as well as increases in the thalamus and cerebellum and a reduction in ventricular volumes.^52^ The recovery trajectories differed between gray and white matter. Regional lobar white matter showed a linear increase across the assessment period. In contrast, regional gray matter showed a nonlinear pattern, with most of the change occurring in the interval between 1 week and 1 month. Even with these increases, the AUD group had lower gray matter volumes than control subjects at the final assessment, with the exception of the frontal lobe. The analyses also identified an interaction of age and smoking, such that with increasing age, the recovery of total cortical and frontal gray matter in individuals who smoked was reduced compared with those who did not smoke. This pattern was consistent with the observed behavioral recovery. The sample was composed primarily of men (88% to 93%, depending on group), precluding study of sex differences.

The researchers also used these data to examine differences between the AUD group and the control group, as well as over time, in brain regions representing core components of the executive control, salience, and emotion networks. These included the dorsal lateral prefrontal cortex (DLPFC), the anterior cingulate cortex (ACC), the orbitofrontal cortex (OFC), insula, amygdala, and hippocampus. The analyses determined that amygdala volumes were not compromised at any point in people with AUD. Also, at the final assessment, the volumes of the ACC, DLPFC, OFC, and insula were equivalent in the AUD and control groups, whereas hippocampal volume remained lower in the AUD group.^53^

A third analysis by this research group explored associations between initial compromise, improvement across time, and treatment outcomes. Comparisons of people with AUD who sustained abstinence versus those who relapsed over the 12 months after treatment showed differences between controls and the two groups even at the initial assessment. People with AUD who eventually relapsed had smaller volumes in three times the number of regions (15/20) as did those who sustained abstinence (5/20). Moreover, among the relapse group, greater gray matter increases during the early weeks of sobriety were associated with longer delays to relapse.^28^

**Figure 3:**
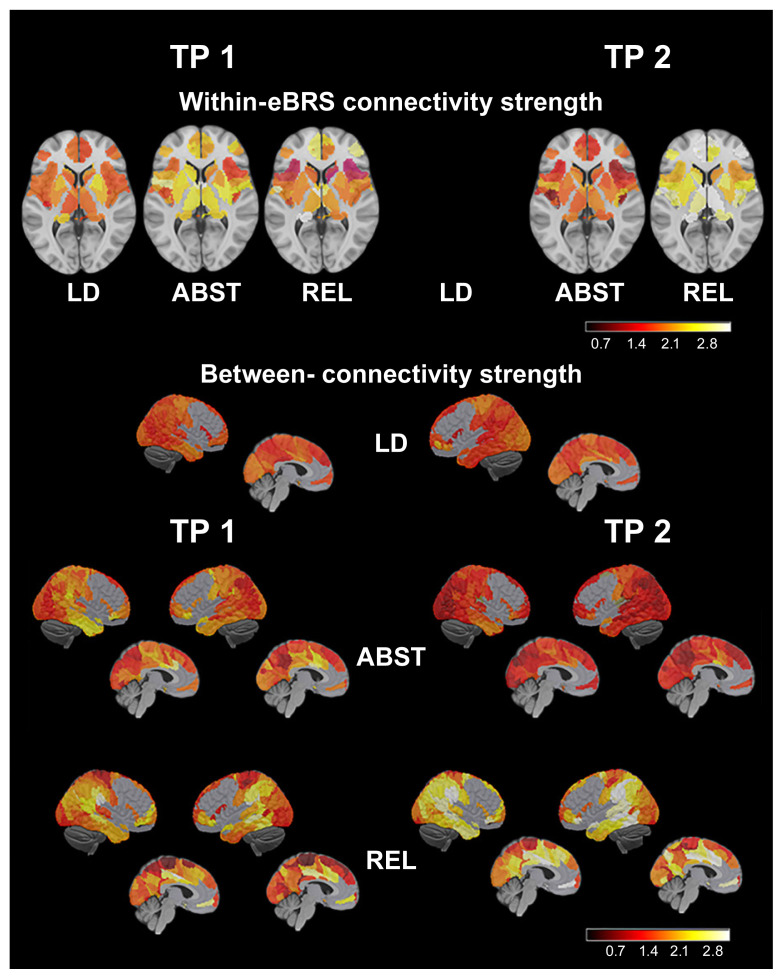
Within-network and between-networks gray matter connectivity (Top) Images on the left show within-extended brain reward system (eBRS) connectivity strength maps for controls (LD) and individuals who are initiating recovery and will either remain abstinent (ABST) or relapse (REL) across the assessment period at their original assessment (TP1=1 month abstinent). Images on the right reflect the degree of connectivity for the ABST and REL groups at TP2 (~ 3 months later). (Bottom) Images show between-networks connectivity strength maps for the LD group at TP1 as well as for the ABST and REL groups at TP1 (left) and TP2 (right). *Note:* Brighter colors and higher numbers on the color bars indicate regions of interest with relatively greater connectivity losses compared to the LD controls (i.e., less connectivity). *Source:* Mueller and Meyerhoff, 2019.^27^ Copyright Society for the Study of Addiction. Reprinted with permission.

Mueller and Meyerhoff^27^ also assessed loss in gray matter and gray matter connectivity within the extended brain reward system—that is, OFC, DLPFC, ACC, insula, striatum, thalami, hippocampi, and amygdala—and its connections with other networks. In longitudinal comparisons at about 1 month abstinent and 3 months later, they found significant resolution in individuals who had sustained abstinence while measures for those who had relapsed remained essentially unchanged (see Figure 3).

Additionally, the research group examined potential genetic modulators of volumetric recovery.^54^ In a study of the Val66Met (rs6265) polymorphism in the brain-derived neurotrophic factor gene (BDNF), they found that between weeks 1 and 5 of abstinence, people homozygous for VAL exhibited increases primarily in gray matter volumes, while heterozygous people (VAL/MET genotype) showed volume increases predominately in white matter. However, the total volume was equivalent for both genotypes at each time point (Note that the sample included no individuals homozygous for MET). Neurocognitive improvement was associated with gray matter increases, but not white matter increases. The same polymorphism also was investigated as a modulator of hippocampal change and neurocognitive function across the first 8 months of abstinence in people with AUD who were homozygous for VAL or carried the MET allele (MET hetero- or homozygous).^55^ Compared with control subjects without AUD, hippocampal volume was lower in the AUD groups at the initial assessment and remained so across all assessments. However, individuals homozygous for VAL were more likely to show hippocampal volume increases across the test interval. Contrary to other reports from this research group,^44^ smoking did not affect initial or recovery measures.

### Neurochemical Change in Recovery

Reduction in neurochemical dysregulation has been examined in a relatively small body of work. Zahr and colleagues^56^ examined levels of NAA, Cho, CR, and glutamate in recently abstinent individuals with AUD (mean days abstinent = 19.6 ± 12.6) and control participants. NAA and Cho levels were inversely affected by pretreatment drinking variables. Of particular interest were findings showing that reduced levels of NAA in the thalamus were found mainly in individuals who would relapse in the 3 months following treatment.

Prisciandaro and colleagues^57^ examined changes in GABA, glutamate, and glutamine by conducting three magnetic resonance spectroscopy sessions across a 1-week monitored abstinence period (i.e., on days 1, 3, and 7) in non–treatment-seeking individuals meeting criteria for an AUD. The participants reported an average of 7.2 drinks/drinking day with an average of 7.8 heavy drinking days (i.e., ≥ 5/4 drinks in a day for men/women, respectively) across the previous 2 weeks. Outcomes showed a significant increase (i.e., normalization) of GABA between scans 1 and 2, without subsequent additional change. In contrast to another report from this research group,^25^ changes in glutamate and glutamine were not robust. Age, which ranged from 21 to 40, did not impact outcomes. There were insufficient numbers of women to permit analysis by sex. The investigators concluded that the difference in outcomes across their studies may be related to sample differences in severity of AUD.

### Summary

The studies reviewed here offer significant insight regarding brain changes in AUD. Unfortunately, women constituted only a small percentage of the study samples, and thus sex differences cannot be adequately explored. Furthermore, much of the published research cited above derives from the efforts of a single research group, and the samples in the separate reports overlap substantially. Given the realities of human neuroimaging studies (i.e., subject costs, selection criteria, resource availability), sample overlap across investigations to ensure study efficiency is not unexpected. While this pattern does not detract from the potential import of the work, it demonstrates the need to replicate the work and expand the samples to allow for evaluation of sex effects.

## INTERVENTION STRATEGIES

An important next question is to what degree the neurobiological and neurobehavioral deficits associated with AUD can be impacted by active interventions. The following sections briefly introduce behavioral and pharmacologic strategies that may facilitate neurobiobehavioral recovery and improve long-term outcomes.^2^ Other approaches, including neuromodulation, are gaining momentum as possible interventions for substance use disorders^58^ but will not be discussed.

### Cognitive Training/Rehabilitation

Examination of cognitive training in AUD has a long history, but few systematic studies were conducted until relatively recently.^2,30^ Performance improvement across training tasks is referred to as “gains,” while the impact of training on additional (untrained) tasks constitutes “transfer of training.” Adaptive training protocols, which adjust to the skill level of the participant, are more efficacious in facilitating training gains and transfer of training, particularly to novel tasks reliant on the trained process (i.e., proximal transfer), than are nonadjusting training protocols.^59^ A key issue is the degree to which training transfers to performance on untrained processes (i.e., distal transfer).

Several examinations applying multi-domain training paradigms reported training-dependent improvements across broad measures. Rupp and colleagues^60^ demonstrated improvements in attention and memory performance among treatment-seeking individuals with AUD. Improvements were observed in several cognitive measures, with multivariate analyses suggesting substantial transfer across tasks. Gamito and colleagues^61^ administered a web-based training to individuals with AUD during inpatient treatment. Results suggested training-associated improvements in composite scores on a battery of executive function tasks. Fals-Stewart and Lam^62^ examined training effects in a 6-month intervention program. Using a training battery engaging diverse neuropsychological domains, they observed transfer to an untrained neuropsychological battery.

In contrast to multi-domain training, contemporary studies often focus on single-domain approaches. Jones and colleagues^63^ investigated training with an inhibitory control task. Despite use of a stop-signal paradigm as both a training and outcome measure, they did not note training-associated improvements. Beyond that study, inhibitory control training remains relatively rare among AUD-focused training examinations, despite its relevance to abstinence maintenance. Other single-domain training approaches have assessed memory improvement. Bell and colleagues^64^ used a training protocol directed at increasing memory capacity among veterans with AUD. They detected training-associated transfer for untrained verbal memory and learning measures. Most of the recent alcohol-related training investigations have used working memory training. Gunn and colleagues^65^ observed proximal transfer on three of six nontrained working memory tasks, two of which continued to display improvement at a 1-month follow-up assessment. Khemiri and colleagues^66^ determined transfer in one verbal working memory task, but no improvement across several additional measures, including alternate working memory tasks. Similarly, Hendershot and colleagues^67^ identified training-associated improvement in a digit span task, but not in three other working memory transfer measures. Snider and colleagues^68^ observed proximal transfer using a “functional” working memory task wherein participants followed a set of sequential object manipulation instructions. In addition to enhanced performance on a functional assessment, this study also noted gains in delay discounting. Although similar assessments of distal transfer remain rare, a recent pilot study suggested that incorporation of emotionally valent stimuli in working memory training may facilitate transfer to social cognition outcomes.^69^

Together, these investigations support assertions that cognitive training may be a useful tool to accelerate cognitive recovery in people with AUD. Proximal transfer has been observed across numerous training studies, while distal transfer has been less commonly examined and, when studied, inconsistently observed. If these interventions are to be effectively utilized, individual and methodological variables contributing to outcome heterogeneity must be systematically interrogated and defined.

### Cognitive Enhancing Medication

Despite substantive efforts directed at drug development for AUD,^70^ improvement in alcohol-associated cognitive deficits has received little consideration as a primary measure of efficacy. Among the FDA-approved medications for AUD, older studies found little impact of naltrexone, subtle decrements resulting from disulfiram, and some putative benefits associated with acamprosate.^71^ A comprehensive review of current AUD-focused drug development efforts is beyond the scope of this article. However, given their demonstrated potential to benefit brain function as evidenced by neurocognitive performance, potential glutamatergic and cholinergic AUD pharmacotherapeutics bear mention.

#### Glutamatergic medications

NMDA glutamate receptors (NMDARs) are integral to learning/memory function, alcohol cue salience, incentive motivation for alcohol use, and mediation of withdrawal-associated neurotoxicity.^72^ Memantine is an FDA-approved, noncompetitive NMDAR channel blocker that may improve AUD-associated outcomes.^73^ In preclinical studies, memantine conferred neuroprotection from withdrawal-associated damage^74^ and ameliorated withdrawal-associated cognitive deficits.^75^ In clinical studies, memantine improved behavioral symptoms and cognitive deficits in alcohol-related dementia.^76^ However, a recent double-blind, placebo-controlled pilot study of treatment-seeking individuals with AUD demonstrated no cognitive benefit.^77^

#### Cholinergic medications

Neuronal nicotinic acetylcholine receptors (nAChRs) are activated by alcohol, facilitating mesolimbic dopamine release.^78^ Animal models indicate a substantive role of nAChRs in mediating both alcohol consumption and relapse behaviors. Taken together with the high prevalence of nicotine use in people with AUD, extant data suggest that nAChR agonists may be useful as putative pharmacotherapies for AUD.^79^ Varenicline is an nAChR agonist with FDA approval for supporting smoking cessation. Varenicline also reduces alcohol consumption among individuals with AUD.^80^ Roberts and McKee^81^ recently examined varenicline-associated cognitive alterations in people with AUD. One week of varenicline administration appeared sufficient to induce dose-dependent improvements in working memory performance and reaction time relative to placebo. At the highest varenicline dose, improvement in working memory performance was associated with larger reductions in alcohol consumption. Galantamine, an nAChR agonist and acetylcholinesterase inhibitor,^82^ appears to reduce relapse severity.^83^ Galantamine appears to improve sustained attention and working memory functions among abstinent individuals with psychostimulant use disorders;^84^ however, its cognitive effects in people with AUD have not been investigated.

### Summary

It is possible that alcohol-related cognitive deficits can be mitigated by behavioral, pharmacologic, or combination therapies. The current body of research is insufficient to draw strong conclusions. Yet, evolving data indicate the promise of systematic research regarding a range of treatment alternatives, both separately and in combination. A critical part of this research must address the fact that extant data cannot fully answer the related question whether these interventions, if successful in improving cognition, impact long-term alcohol use patterns. Thus, the path forward requires a highly programmatic approach.

## CONCLUSIONS, LIMITATIONS, AND FUTURE DIRECTIONS

A large body of research has examined the persistence of alcohol-related neurobiological and behavioral compromise after detoxification. Encouraging data, acquired across decades of research, have revealed a reduction in impairment following the initiation of abstinence. Significant neurobehavioral improvement has been observed in the early weeks of abstinence, with some continuing recovery in later months. For some measures, deficits are mitigated, but measurable compromise persists compared with healthy controls. Similar conclusions can be drawn regarding improvement in neurophysiological measures, brain volume, neurochemistry, white matter integrity, and brain network integration/activation. One of the most striking outcomes is the substantial research suggesting that improvement is contingent on sustained abstinence. Increased age frequently is associated with less effective recovery. Limited data are available regarding sex differences, with inconsistent results, and still fewer studies have considered the interaction of age and sex. Finally, it is important to keep in mind that adaptive behavior change may occur even in the absence of substantial structural or neurophysiological “recovery” compared with initial brain or behavior compromise. These adaptations may be mediated by the engagement of compensatory mechanisms/processes, such as sacrificing response speed to enhance accuracy or engaging alternate or additional brain areas. This issue remains largely understudied in the context of AUD recovery.^4^

One strength of current research is the ability to probe the interrelationships of structure and function. As shown in previous sections, developing science extends and clarifies earlier conclusions and affords the opportunity to disentangle neurobiobehavioral processes that may differentially contribute to improvement. These advances promote both scientific and clinical progress. For example, Galandra and colleagues^23^ demonstrated that alcohol-related deficits in aspects of executive functions may be mediated by dysregulation in the salience network. Based on current understanding of the functions and underlying structure of the salience network, this finding is consistent with cognitive frameworks that emphasize failures in active ignoring as a core component of alcohol-related executive function deficits. Together, the neurobiological and behavioral data provide a rationale for the testable hypothesis that improving the ability to ignore irrelevant stimuli (i.e., enhancing active ignoring skills) may be a useful target for behavioral interventions. Similarly, existing research suggests that programmatic integration of cognitive training interventions and cognitive enhancing medications, as well as evolving technologies such as neuromodulation, may accelerate cognitive recovery and ultimately long-term outcomes.

Despite the promise of existing data, there are notable limitations. First, although there are notable exceptions, post-treatment outcomes are often ascertained across a few weeks or months. Thus, long-term trajectories remain understudied. Second, the complexity of conducting systematic longitudinal studies is daunting. Thus, investigators must take full advantage of available data, resources, and volunteers. The result is that a limited sample may contribute to numerous, interdependent studies. Consequently, the findings from a body of work where the supporting studies are populated by overlapping samples may not be generalizable. Third, as noted in the introduction, individual differences are understudied. To the extent possible, this review has discussed the influence of age and sex. However, other less immediately obvious individual variables, such as nutritional status, also are pertinent,^85^ but were beyond the scope of this review. Finally, as summarized above, sustained abstinence was required to show improvement across many of these studies. Moreover, participants in the large majority of these studies were individuals seeking treatment, often in inpatient or intensive outpatient facilities and typically meeting criteria for more severe AUD. Thus, the findings described here do not address outcomes among individuals who meet criteria for AUD but who engage in non–abstinence-based treatment or initiate recovery without employing formal treatment programs. A person’s selected pathway to recovery is, no doubt, influenced by significant environmental and individual variables that may, themselves, be associated with differential baseline compromise and recovery trajectories. Therefore, all efforts to advance science and practice must take into consideration alternative definitions of “recovery.”^86^
